# Postural influences on sweating: exploring the effects of gravity and pressure

**DOI:** 10.1186/2046-7648-4-S1-A154

**Published:** 2015-09-14

**Authors:** Norikazu Ohnishi, Sean R Notley, Joonhee Park, Kyoko Tagami, Catriona A Burdon, Elizabeth A Taylor, Nigel AS Taylor

**Affiliations:** 1 Centre for Human and Applied Physiology, School of Medicine, University of Wollongong, Wollongong, Australia

## Introduction

The distribution of thermal sweating is neither uniform nor does it commence simultaneously at all sites. One reason for this variability may be associated with gravitational influences. That is, localised and posture-dependent compression of tissues containing pressure-sensitive receptors is believed to inhibit sweating from the compressed and ipsilateral sites, whilst enhancing secretion from contralateral surfaces [1]. To evaluate the possibility that local sweat rates might be influenced by gravity, it is necessary to test subjects with and without gravitational loading. This can be achieved by using water immersion to simulate zero gravity, and this experimental model was used for this pilot investigation.

## Methods

Eight males (blindfolded) were first exposed to a supine, resting air exposure (control: 28°C, 60% relative humidity) wearing a slowly heated, water-perfusion suit (40 ^o^, 45 ^o^, 50°C), and then to a supine water immersion (38°C). Local sweat rates were measured using ventilated capsules (3.16 cm^2^; capacitance hygrometry) positioned at two ventral surfaces (forehead, lower chest) and on the dorsal surfaces of the pronated hand and foot. Sweating was tracked until steady states were approximated across all sites.

## Results

Due to the nature of these thermal stimuli, similar, but not identical, auditory canal temperatures were evoked, with immersion producing significantly greater thermal loading: 36.6 ^o ^versus 36.9 °C. To adjust for this thermal bias, data were analysed at similar auditory canal temperatures (36.6 ^o ^[control] and 36.7 °C, respectively), although matching was imperfect, resulting in sweating being evaluated only during transient steady states. When gravitational compression of the dorsal body surfaces was minimised, 72 % of the within-subject and within-site comparisons revealed lower, temperature-dependent sweat rates, even though the matched core temperatures were higher during immersion. This was evident at the forehead, chest and hand in six of eight subjects. Thus, when expressed as sudomotor ratios for each individual (control/immersion) and then averaged across subjects, sweating from the non-compressed surfaces was >2.5-fold (forehead), >9.4-fold (chest), >6.0-fold (hand) and >6.4-fold (foot) higher during the control trial. However, the absolute sweat rate differences were not significant (*P *> 0.05).

## Discussion

Whilst these observations were qualitatively consistent with a neural, hemihidrotic effect [1], it is possible this experiment was under powered, and lacked sufficient control for its more complete expression. Furthermore, since we used partial immersion and not total submersion, then some gravity-induced compression remained. Therefore, whilst it is tempting to speculate that these observations were due to the influence of a buoyancy-associated reduction in skin pressure, which attenuated hemihidrosis, it is probable that multiple mechanisms are involved in the modulation of sweating activity during postural and gravitational variations.
